# Non-contrast MRI features of mucin plugs and mural nodules in pancreatic cystic lesions

**DOI:** 10.1007/s11604-026-01975-x

**Published:** 2026-03-31

**Authors:** Hideyuki Fukui, Atsushi Nakamoto, Hiromitsu Onishi, Takashi Ota, Yasunari Fukuda, Toru Honda, Sakiko Ueno, Feier Ding, Masahiro Umezu, Daisaku Yamada, Hidetoshi Eguchi, Noriyuki Tomiyama

**Affiliations:** 1 Department of Diagnostic and Interventional Radiology, The University of Osaka Graduate School of Medicine, D1, 2-2, Yamadaoka, Suita, Osaka 565-0871 Japan; 2 Department of Gastroenterological Surgery, The University of Osaka Graduate School of Medicine, Suita, Japan; 3 Department of Surgery, Osaka International Cancer Institute, Osaka, Japan

**Keywords:** Pancreatic cystic lesions, Intraductal papillary mucinous neoplasm, Non-contrast MRI, Target sign, Mural nodule, Mucin plug

## Abstract

**Purpose:**

Non-contrast MRI characteristics can help differentiate mucin plugs from mural nodules in pancreatic cystic lesions. To identify MRI features that differentiate non-enhancing lesions from true mural nodules in pancreatic cystic lesions.

**Materials and methods:**

We retrospectively evaluated 90 patients with pancreatic cystic lesions containing 101 nodular components (62 non-enhancing, 22 benign enhancing, 17 high-risk). Two experienced radiologists independently assessed multiple non-contrast MRI characteristics, including signal-intensity patterns, shape, and their cyst-wall relationship. The reference standard was the contrast-enhanced pattern, with histopathology for resected specimens or minimum 2-year stability for presumed mucin plugs. Diagnostic performance was evaluated in binary logistic regression models.

**Results:**

Only the non-enhancing lesions (41.9%) showed the “target sign” (layered appearance due to different central- and peripheral-portion signal intensities), providing 100% specificity for identifying non-enhancing lesions. The non-enhancing lesions were predominantly oval-shaped (93.5%) with posterior-wall attachment (85.5%), whereas enhancing lesions showed a complex morphology. On diffusion-weighted imaging (DWI), 93.5% of the non-enhancing lesions showed low signal intensity and 95.2% showed high apparent diffusion coefficient** (**ADC) values, contrasting with high DWI signal (82.4%) and low ADC values (82.4%) in the high-risk lesions. A multiparameter model achieved excellent diagnostic performance with an area under the curve of 0.97 for differentiating non-enhancing from enhancing lesions and 0.92 for distinguishing non- or benign enhancing from high-risk lesions.

**Conclusions:**

Non-contrast MRI features, particularly the target sign, shape characteristics, and cyst-wall relationship can reliably differentiate mucin plugs from true mural nodules in pancreatic cystic lesions, potentially reducing unnecessary invasive procedures.

**Supplementary Information:**

The online version contains supplementary material available at 10.1007/s11604-026-01975-x.

## Introduction

Pancreatic cystic lesions, particularly intraductal papillary mucinous neoplasms (IPMNs), have become increasingly prevalent with the widespread use of high-resolution cross-sectional imaging [1–3]. These lesions occur along a spectrum from benign to malignant, posing a significant diagnostic challenge [4, 5]. Accurate characterization of pancreatic cystic lesions is crucial for determining appropriate management strategies and optimizing patient outcomes. Among the imaging features of pancreatic cysts, mural nodules are a high-risk predictor of malignancy in current international guidelines [4, 5]. Therefore, detection and characterization of mural nodules is critical for risk stratification of pancreatic cystic lesions, and accurate differentiation between true mural nodules and mucin plugs is essential for appropriate clinical management [4, 5]. Mural nodules are true neoplastic cyst-wall growths, with a higher risk of malignancy and often requiring surgery [6, 7]. Mucin plugs are an accumulation of viscous mucoid material without neoplastic potential [8–11].

Currently, differentiating between true mural nodules and mucin plugs fundamentally relies on assessing enhancement patterns on contrast-enhanced MRI (CE-MRI) or contrast-enhanced CT (CE-CT). True mural nodules typically demonstrate enhancement after contrast administration, whereas mucin plugs remain non-enhancing [12, 13]. However, clinically, many centers use non-contrast MRI protocols for surveillance because of examination costs [14, 15], potential adverse effects of gadolinium-based contrast agents [16], and the need for repeated examinations over extended periods, among other factors [13, 17, 18].

Recent years have witnessed a paradigm shift toward abbreviated non-contrast MRI protocols for pancreatic cyst surveillance. Some institutions have adopted abbreviated protocols, achieving 70–85% scanning-time reductions while maintaining diagnostic accuracy [14, 15, 18–20]. These protocols typically use T2-weighted imaging (T2WI) and 3D-magnetic resonance cholangiopancreatography (MRCP) sequences, omitting contrast-enhanced sequences.

Therefore, with non-contrast MRI surveillance becoming increasingly common, differentiation between mural nodules and mucin plugs without contrast administration would be highly desirable to reduce examination costs, eliminate contrast-related risks, and streamline surveillance protocols while maintaining diagnostic accuracy. However, no studies using non-contrast MRI features alone have determined if differentiating between mural nodules and mucin plugs is possible.

This study aimed to systematically evaluate non-contrast MRI characteristics useful for differentiating mucin plugs from mural nodules in pancreatic cystic lesions to enhance the clinical utility of abbreviated surveillance protocols and improve patient care to provide less costly healthcare.

## Materials and methods

This study was approved by the Institutional Review Board at Osaka University Hospital (Approval Number: [24405]), and informed consent was waived for this retrospective study.

### Patients

We searched reports of upper-abdominal MRI examinations at our institution between January 2006 and December 2024 for the following keywords: “mucin,” “debris,” “solid component,” or “mural nodule,” yielding 11,835 reports; however, only 163 positive cases were identified. The inclusion criteria were 1) the presence of mucin, solid components, or mural nodules within pancreatic cysts and 2) the availability of contrast-enhanced imaging evaluation (CE-MRI or, alternatively, contrast-enhanced endoscopic ultrasound (CE-EUS) (n = 16) or CE-CT (n = 6) when MRI was not feasible). Ultimately, 118 cases were eligible for the study.

**Fig. 1:**
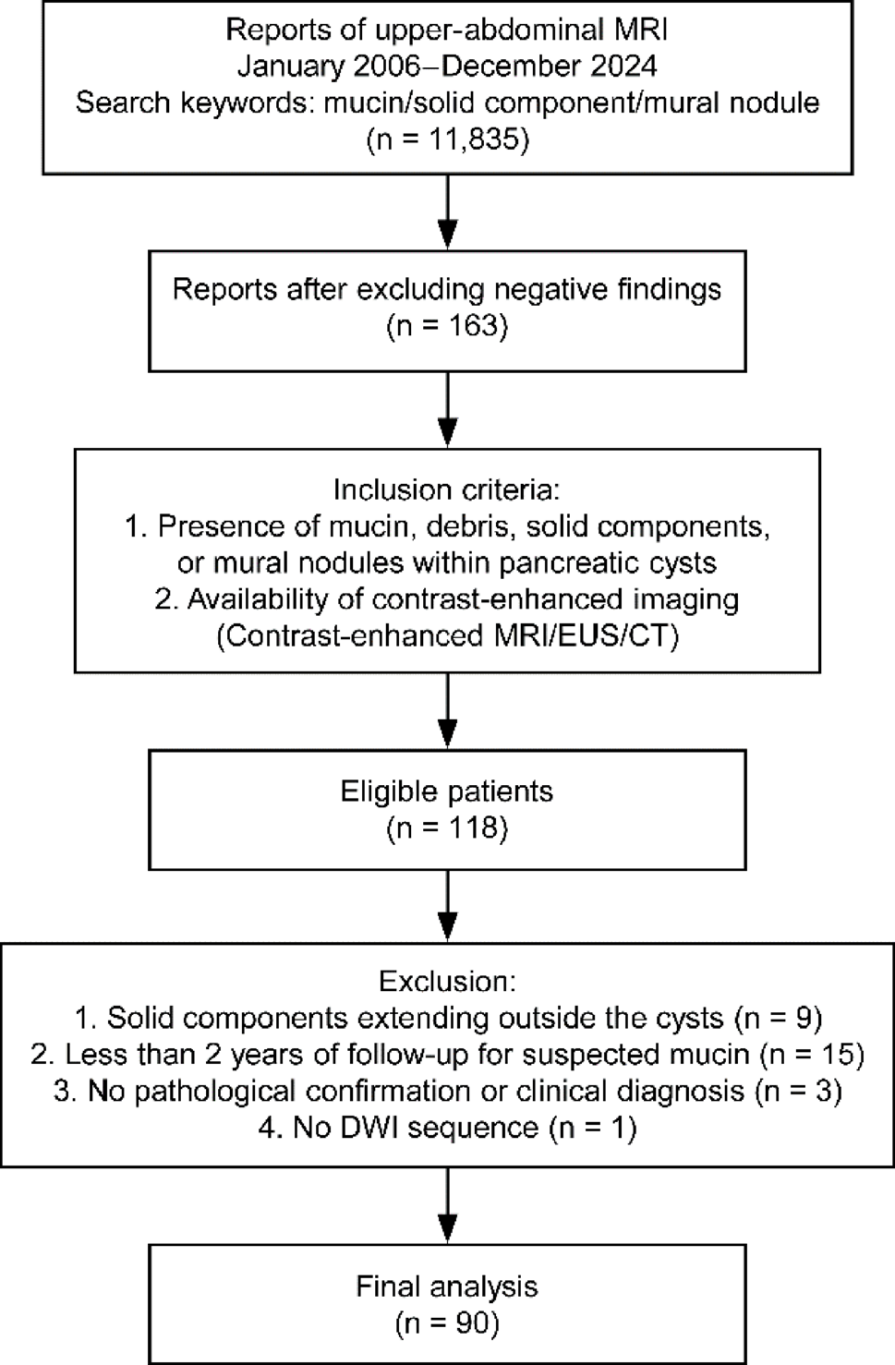
Flowchart of patient inclusion

We excluded cases with solid components extending outside the cystic lesion (n = 9), as determined by consensus between two experienced radiologists (H.F. and T.O., both with 14 years of abdominal radiology experience). We also excluded patients with suspected mucin plug showing no changes during follow-up but who had < 2 years of follow-up (n = 15), patients with enhanced solid components but without pathological confirmation or clinical diagnosis (n = 3), and patients without diffusion-weighted imaging (DWI) sequences (n = 1). Finally, 90 patients were included (Fig. 1).

### Imaging protocol

Standardized protocols were used to perform multimodality imaging. MRI was performed on 3.0-T systems (GE Healthcare, Philips Healthcare, Canon Medical Systems) and a 1.5-T system (Philips Healthcare) with phased-array body coils using comprehensive protocols, including fat-suppressed T1-weighted imaging (T1WIfs), T2WI, DWI, and contrast-enhanced imaging sequences. CT was performed on 64–320-detector-row systems (Aquilion series, Canon Medical Systems, Tokyo, Japan) with intravenous iodinated contrast agents (iopamidol, iohexol, ioversol, or iomeprol) following a multiphasic protocol. CE-EUS was performed using curvilinear echoendoscopes with Sonazoid (Daiichi Sankyo, Tokyo, Japan) as the contrast agent. Detailed acquisition parameters and reconstruction protocols for MRI are presented in Supplemental Table E1. For patients without CE-MRI who required alternative imaging for enhancement evaluation, the mean interval between non-contrast MRI and CE-EUS examinations was 44.8 days (n = 16), and the mean interval between non-contrast MRI and CE-CT examinations was 134.0 days (n = 6).

**Table 1:** Demographic and Clinical Characteristics of the Study Population

Characteristic	Non-enhancing Lesions (n = 51)	Benign Enhancing Lesions (n = 22)	High-Risk Lesions (n = 17)	Total (n = 90)
*Sex*				
Female	23 (45.1%)	10 (45.5%)	6 (35.3%)	39 (43.3%)
Male	28 (54.9%)	12 (54.5%)	11 (64.7%)	51 (56.7%)
*Diabetes Mellitus*				
No	50 (98.0%)	15 (68.2%)	15 (88.2%)	80 (88.9%)
Yes	1 (2.0%)	7 (31.8%)	2 (11.8%)	10 (11.1%)
*Obesity (BMI* ≥ *25)*				
No	41 (80.4%)	19 (86.4%)	15 (88.2%)	75 (83.3%)
Yes	10 (19.6%)	3 (13.6%)	2 (11.8%)	15 (16.7%)
*Alcohol Consumption*				
No	38 (74.5%)	16 (72.7%)	13 (76.5%)	67 (74.4%)
Yes	13 (25.5%)	6 (27.3%)	4 (23.5%)	23 (25.6%)
*Smoking History*				
No	34 (66.7%)	15 (68.2%)	11 (64.7%)	60 (66.7%)
Yes	17 (33.3%)	7 (31.8%)	6 (35.3%)	30 (33.3%)
CA19-9 (U/mL)	20.05 ± 23.11	18.67 ± 15.32	52.64 ± 102.76	25.54 ± 47.96
CEA (ng/mL)	2.78 ± 2.35	2.36 ± 1.29	3.59 ± 3.47	2.81 ± 2.38
Subcategory IPMN with LGD SCN NEN MCN IPMN with WF/HRS, stable > 5 years IPMN with HGD IPMN with IC		16 1 1 1 3	10 7	

Values are presented as n (%) for categorical variables or mean ± SD for continuous variables. Abbreviations: BMI, Body Mass Index; CA19-9, carbohydrate antigen 19–9; CEA, carcinoembryonic antigen; HGD, high-grade dysplasia; HRS, high-risk stigmata; IC, invasive carcinoma; IPMN, intraductal papillary mucinous neoplasm; LGD, low-grade dysplasia; MCN, mucinous cystic neoplasm; NEN, neuroendocrine neoplasm; SCN, serous cystic neoplasm; WF, worrisome features

### Image analysis and data collection

The final nodule classification was primarily based on enhancement patterns. Nodules were first differentiated by enhancement on CE-MRI. For cases without CE-MRI, CE-EUS was used, but if not available, CE-CT was used to determine enhancement status. For surgically resected specimens, histopathological confirmation was the reference standard. For suspected non-enhancing lesions (NELs) (presumed mucin plugs or debris) without surgical confirmation, stability for ≥ 2 years or disappearance during follow-up was required to confirm their benign nature. According to these criteria, nodules were classified into three groups: 1) NELs (including mucin plugs and debris); 2) benign enhancing lesions (BELs), including IPMNs with low-grade dysplasia, serous cystic neoplasms, neuroendocrine neoplasms (NENs), mucinous cystic neoplasms, and IPMNs with worrisome features or high-risk stigmata that remained stable during > 5 years of follow-up; and 3) high-risk lesions (HRLs), defined as lesions with high-grade dysplasia or invasive carcinoma.

The same two radiologists independently evaluated all non-contrast images while blinded to the final diagnosis and clinical information. Each reviewer systematically assessed multiple characteristics of both the cystic lesions and any associated nodular components. Each reader’s assessments were analyzed independently, and the results from both readers are presented separately.

For each nodular component within the pancreatic cystic lesions, the radiologists measured the maximum nodule diameter in millimeters and classified nodule shape as oval, irregular, or villous. For analysis, these shape classifications were later re-categorized as oval or non-oval (including both irregular and villous patterns) to optimize statistical modeling. Each nodule’s position relative to the cyst wall was categorized as posterior, anterior, or lateral attachment, which were subsequently simplified to posterior or other attachment for statistical analysis, based on the clinical observation that NELs tend to settle in a gravity-dependent position.

The radiologists documented the presence or absence of a target sign, a layered appearance with different signal intensities between the central and peripheral portions of the lesion. This pattern has been previously described in EUS studies as a characteristic feature of mucin plugs [9–11, 21] and we sought to identify its MRI correlate on various sequences. For an absent target sign, the nodule’s signal intensity was classified as high, intermediate, or low compared with normal pancreatic parenchyma on T2WI and T1WIfs. Signal intensity on DWI was qualitatively assessed as high, intermediate, or low, whereas apparent diffusion coefficient** (**ADC) values were relatively assessed as high, intermediate, or low compared with normal pancreatic parenchyma.

For the cystic lesions themselves, the maximum cyst diameter was measured in millimeters on the image showing the largest cross-sectional area. Cyst morphology was either unilocular or multilocular. The main pancreatic duct (MPD) diameter (mm) was measured at the widest visualized portion. Additional recorded parameters included the number of nodules within each cyst and the cyst location (pancreatic head, body, or tail).

Patient demographic and clinical data were collected from electronic medical records, including age at examination, sex, obesity (body mass index ≥ 25 kg/m^2^), smoking history, alcohol consumption history, diabetes presence, and tumor-marker levels (carbohydrate antigen 19-9 [CA19-9] and carcinoembryonic antigen [CEA]). When multiple cysts or nodules were present in one patient, each was analyzed individually with corresponding patient demographic data applied to each observation. This approach was based on the clinical rationale that characteristics of different nodules within the same patient could be considered independent for analytical purposes.

### Statistical analysis

Patient-, cyst-, and nodule-level characteristics are reported using means and standard deviations for continuous variables and frequencies and proportions for categorical variables. These summary statistics are intended to describe the data observed in this study. In line with the STROBE statement for reporting observational studies [22], no statistical tests were performed for these descriptive variables.

Cohen’s kappa coefficient was used to assess inter-observer agreement between radiologists for MRI findings and between MRI and EUS findings. The diagnostic performance of the target sign for NELs was evaluated by calculating sensitivity, specificity, positive predictive value (PPV), and negative predictive value (NPV). The target sign presence was defined as positive in this study. Accordingly, sensitivity was defined as the proportion of NELs exhibiting the target sign, specificity, as the proportion of enhancing lesions not exhibiting the target sign, PPV as the probability that a lesion with the target sign was non-enhancing, and NPV as the probability that a lesion without the target sign was enhancing. For calculating the PPV and NPV, the prevalence of enhancing nodules was assumed to be 40% on the basis of previous studies [9–11].

As the primary analysis, Firth logistic regression to differentiate NELs from enhancing lesions was used to construct a multivariable model for each reader, and receiver operating characteristic (ROC) curves with corresponding area under the curves (AUCs) were generated. Firth’s correction was intended to address complete separation between the target sign and diagnostic outcome. The multivariable model also included nodule diameter, shape, wall relationship, cyst diameter and morphology, MPD diameter, and age selected on the basis of clinical relevance. As a secondary analysis, a similar approach was also used to classify lesions as NELs/BELs or HRLs. R version 4.4.3 (R Foundation for Statistical Computing, Vienna, Austria) was used to perform all analyses.

## Results

### Patient and lesion characteristics

Ninety patients with pancreatic cystic lesions were analyzed (Table 1), comprising 96 cysts and 101 nodules; 62 were NELs, 22 were BELs, and 17 were HRLs. The prevalence of the enhancing nodules was 38.6% (39/101), consistent with the assumed prevalence of 40% based on previous studies.

Gender, obesity, alcohol consumption, and smoking history were similar across the three patient groups. The prevalence of diabetes seemed to differ across groups (31.8% in BELs; 11.8% in HRLs; 2.0% in NELs). CA19-9 levels also appeared to differ across groups (52.64 ± 102.76 U/mL in HRLs; 18.67 ± 15.32 U/mL in BELs; 20.05 ± 23.11 U/mL in NELs).

### Morphological features

The maximum cyst diameter increased from NEL (18.23 ± 13.36 mm) to BEL (32.41 ± 17.20 mm) to HRL (42.29 ± 17.16 mm). Similarly, the nodule mean diameter was larger for HRL (19.06 ± 14.92 mm) than for BEL (9.59 ± 3.89 mm) and NEL (6.60 ± 2.87 mm).

Cyst morphology showed differences between the groups, with NEL predominantly unilocular (91.2%) and enhancing lesions mostly multilocular (72.7% of benign, 82.4% of HRL). All HRL cases showed MPD communication, 86.4% had BELs, and 73.7% had NEL (Table 2). For cyst-related assessments, evaluations by reader 2 were in complete agreement with those by reader 1 (Supplemental Table E2). All kappa coefficients were equal to 1.

**Table 2:** Cyst Characteristics by Diagnostic Group (Reader 1)

Parameter	Category/Measurement	Non-enhancing Lesions	Benign Enhancing Lesions	High-Risk Lesions	Total
Maximum Cyst Diameter (mm)		18.23 ± 13.36	32.41 ± 17.20	42.29 ± 17.16	25.74 ± 17.70
Cyst Morphology	Unilocular	52 (91.2%)	6 (27.3%)	3 (17.6%)	61 (63.5%)
	Multilocular	5 (8.8%)	16 (72.7%)	14 (82.4%)	35 (36.5%)
	**Subtotal**	**57 (100.0%)**	**22 (100.0%)**	**17 (100.0%)**	**96 (100.0%)**
MPD Communication	No	15 (26.3%)	3 (13.6%)	0 (0.0%)	18 (18.8%)
	Yes	42 (73.7%)	19 (86.4%)	17 (100.0%)	78 (81.2%)
	**Subtotal**	**57 (100.0%)**	**22 (100.0%)**	**17 (100.0%)**	**96 (100.0%)**
Cyst Location	Head	21 (36.8%)	11 (50.0%)	13 (76.5%)	45 (46.9%)
	Body	9 (15.8%)	4 (18.2%)	1 (5.9%)	14 (14.6%)
	Tail	27 (47.4%)	7 (31.8%)	3 (17.6%)	37 (38.5%)
	**Subtotal**	**57 (100.0%)**	**22 (100.0%)**	**17 (100.0%)**	**96 (100.0%)**
T2WI/Balanced/MRCP	High	57 (100.0%)	22 (100.0%)	17 (100.0%)	96 (100.0%)
	Intermediate	0 (0.0%)	0 (0.0%)	0 (0.0%)	0 (0.0%)
	Low	0 (0.0%)	0 (0.0%)	0 (0.0%)	0 (0.0%)
	**Subtotal**	**57 (100.0%)**	**22 (100.0%)**	**17 (100.0%)**	**96 (100.0%)**
T1WIfs	High	1 (1.8%)	1 (4.5%)	0 (0.0%)	2 (2.1%)
	Intermediate	0 (0.0%)	0 (0.0%)	0 (0.0%)	0 (0.0%)
	Low	56 (98.2%)	21 (95.5%)	17 (100.0%)	94 (97.9%)
	**Subtotal**	**57 (100.0%)**	**22 (100.0%)**	**17 (100.0%)**	**96 (100.0%)**
DWI	High	1 (1.8%)	0 (0.0%)	0 (0.0%)	1 (1.0%)
	Intermediate	0 (0.0%)	1 (4.5%)	0 (0.0%)	1 (1.0%)
	Low	56 (98.2%)	21 (95.5%)	17 (100.0%)	94 (97.9%)
	**Subtotal**	**57 (100.0%)**	**22 (100.0%)**	**17 (100.0%)**	**96 (100.0%)**
ADC	High	57 (100.0%)	22 (100.0%)	17 (100.0%)	96 (100.0%)
	Intermediate	0 (0.0%)	0 (0.0%)	0 (0.0%)	0 (0.0%)
	Low	0 (0.0%)	0 (0.0%)	0 (0.0%)	0 (0.0%)
	**Subtotal**	**57 (100.0%)**	**22 (100.0%)**	**17 (100.0%)**	**96 (100.0%)**

Values are presented as n (%) for categorical variables or mean ± SD for continuous variables. Abbreviations: MPD, main pancreatic duct; MRCP, magnetic resonance cholangiopancreatography; T2WI, T2-weighted imaging; T1WIfs, fat-suppressed T1-weighted imaging; DWI, diffusion-weighted imaging; ADC, apparent diffusion coefficient

### MRI signal characteristics of nodular components

The target sign on T2WI/Balanced/MRCP was exclusively observed in NEL (41.9%) and absent in all enhancing lesions. On T2WI/Balanced/MRCP, 76.5% of HRL, 36.4% of BEL, and 9.7% of NEL showed intermediate signal intensity. On DWI, 93.5% of NEL showed low signal intensity, whereas 82.4% of HRL showed high signal intensity. On ADC maps, 95.2% of NEL showed high values, and 82.4% showed low values.

The shape distribution indicated differences between groups: 93.5% of NEL were oval, but irregular and villous shapes were more common in enhancing lesions. Regarding the cyst-wall relationship, 85.5% of NEL had posterior attachment, which is consistent with a gravitational effect, whereas enhancing lesions predominantly showed non-posterior attachment (Table 3). The assessments by reader 2 tended to be similar to these findings (Supplemental Table E3). The inter-reader kappa coefficients for T2WI/Balanced/MRCP, T1WIfs, DWI, and ADC were 0.95, 1.00, 0.96, and 0.93, respectively.

**Table 3:** Nodule Characteristics by Diagnostic Group (Reader 1)

Parameter	Category/Measurement	Non-Enhancing Lesions	Benign Enhancing Lesions	High-Risk Lesions	Total
Nodule Diameter (mm)	Mean ± SD	6.60 ± 2.87	9.59 ± 3.89	19.06 ± 14.92	9.35 ± 8.04
T2WI/Balanced/MRCP	High	0 (0.0%)	2 (9.1%)	0 (0.0%)	2 (2.0%)
	Intermediate	6 (9.7%)	8 (36.4%)	13 (76.5%)	27 (26.7%)
	Low	30 (48.4%)	12 (54.5%)	4 (23.5%)	46 (45.5%)
	Target	26 (41.9%)	0 (0.0%)	0 (0.0%)	26 (25.7%)
	**Subtotal**	**62 (100.0%)**	**22 (100.0%)**	**17 (100.0%)**	**101 (100.0%)**
T1WIfs	High	1 (1.6%)	0 (0.0%)	0 (0.0%)	1 (1.0%)
	Intermediate	1 (1.6%)	3 (13.6%)	4 (23.5%)	8 (7.9%)
	Low	58 (93.5%)	19 (86.4%)	13 (76.5%)	90 (89.1%)
	Target	2 (3.2%)	0 (0.0%)	0 (0.0%)	2 (2.0%)
	**Subtotal**	**62 (100.0%)**	**22 (100.0%)**	**17 (100.0%)**	**101 (100.0%)**
DWI	High	2 (3.2%)	12 (54.5%)	14 (82.4%)	28 (27.7%)
	Intermediate	2 (3.2%)	2 (9.1%)	1 (5.9%)	5 (5.0%)
	Low	58 (93.5%)	8 (36.4%)	2 (11.8%)	68 (67.3%)
	**Subtotal**	**62 (100.0%)**	**22 (100.0%)**	**17 (100.0%)**	**101 (100.0%)**
ADC	High	59 (95.2%)	10 (45.5%)	3 (17.6%)	72 (71.3%)
	Intermediate	0 (0.0%)	1 (4.5%)	0 (0.0%)	1 (1.0%)
	Low	3 (4.8%)	11 (50.0%)	14 (82.4%)	28 (27.7%)
	**Subtotal**	**62 (100.0%)**	**22 (100.0%)**	**17 (100.0%)**	**101 (100.0%)**
Shape	Oval	58 (93.5%)	7 (31.8%)	3 (17.6%)	68 (67.3%)
	Irregular	4 (6.5%)	9 (40.9%)	8 (47.1%)	21 (20.8%)
	Villous	0 (0.0%)	6 (27.3%)	6 (35.3%)	12 (11.9%)
	**Subtotal**	**62 (100.0%)**	**22 (100.0%)**	**17 (100.0%)**	**101 (100.0%)**
Relationship to the Wall	Posterior attachment	53 (85.5%)	6 (27.3%)	5 (29.4%)	64 (63.4%)
	Non-posterior attachment	5 (8.1%)	15 (68.2%)	12 (70.6%)	32 (31.7%)
	Non-attached	4 (6.5%)	1 (4.5%)	0 (0.0%)	5 (5.0%)
	**Subtotal**	**62 (100.0%)**	**22 (100.0%)**	**17 (100.0%)**	**101 (100.0%)**

Values are presented as n (%) for categorical variables or mean ± SD for continuous variables. Abbreviations: T2WI, T2-weighted imaging; MRCP, magnetic resonance cholangiopancreatography; T1WIfs, fat-suppressed T1-weighted imaging; DWI, diffusion-weighted imaging; ADC, apparent diffusion coefficient

### Diagnostic performance and ROC analysis

The two readers were in perfect agreement in the target-sign assessment; the sensitivity, specificity, PPV, and NPV of the target sign in the differentiating NELs were 41.9%, 100%, 100%, and 53.4%, respectively. Inter-observer agreement between the radiologists for all MRI findings (Target/High/Intermediate/Low) was excellent (κ = 0.95), and MRI/EUS findings agreement was also good (κ = 0.71).

**Fig. 2:**
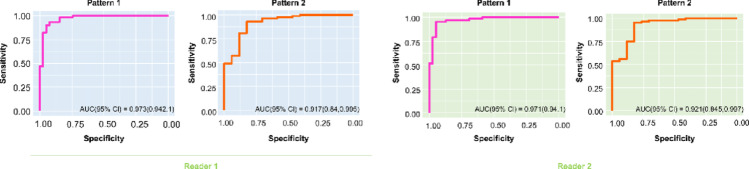
Receiver operating characteristic curves comparing diagnostic performance for Pattern 1 (differentiating non-enhancing from enhancing lesions, pink) and Pattern 2 (differentiating non- or benign enhancing lesions from high-risk lesions, orange) The multiparameter model incorporating nodule diameter, shape, wall relationship, cyst features, and target sign presence achieved excellent diagnostic accuracy (AUC values of 0.97 and 0.92 for Patterns 1 and 2, respectively)

Figure 2 shows the ROC curves based on the multivariable models. For differentiating NELs (Pattern 1), the AUC was 0.973 [95% confidence intervals (CI) 0.942–1.000] for Reader 1 and 0.971 (95% CI 0.940–1.000) for Reader 2. For differentiating NELs/BELs (Pattern 2), the AUC was 0.917 (95% CI 0.840–0.995) for Reader 1 and 0.921 (95% CI 0.845–0.997) for Reader 2. Based on Youden’s Index, the sensitivity, specificity, PPV, and NPV for differentiating NELs in the multivariable model were 0.935, 0.923, 0.948, and 0.905 for Reader 1, and 0.952, 0.949, 0.965, and 0.929 for Reader 2, respectively, demonstrating substantial improvement in sensitivity and NPV versus using the target sign alone. Shape was the only variable in the model for which the readers’ evaluation results differed.

**Fig. 3:**
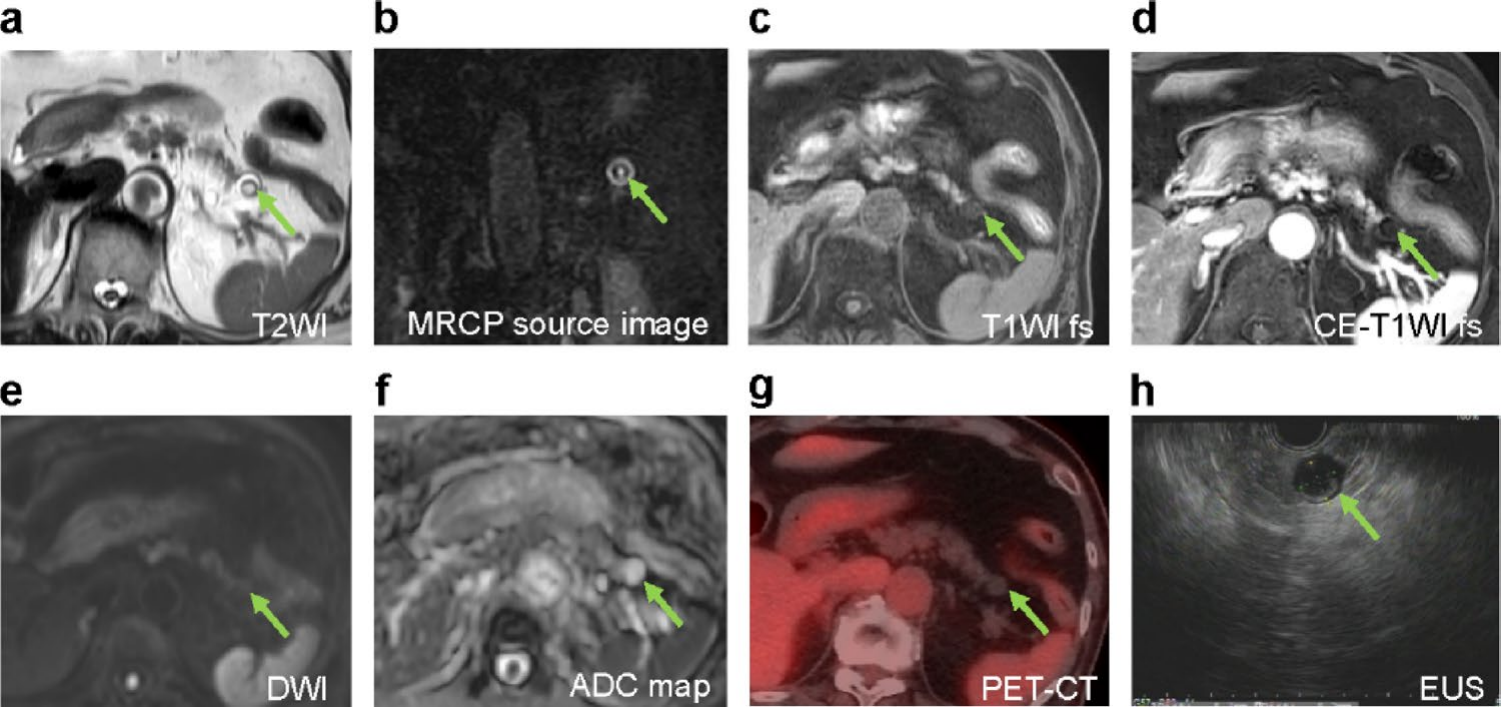
Non-enhancing lesion in an 83-year-old female **a** T2-weighted imaging showing a cystic lesion with a gravity-dependent nodular component (green arrow) exhibiting a target sign. **b** Magnetic resonance cholangiopancreatography source image demonstrating the characteristic target sign. **c** Fat-suppressed T1-weighted imaging showing hypointense signal. **d** Contrast-enhanced fat-suppressed T1-weighted imaging showing no enhancement. **e** Diffusion-weighted imaging showing low signal intensity.** f** Apparent diffusion coefficient map showing high values. **g** Positron-emission tomography-computed tomography showing no significant ^18^F-fluorodeoxyglucose uptake. **h** Endoscopic ultrasonography confirming the target sign appearance. Abbreviations: ADC, apparent diffusion coefficient; CE-T1WI, contrast-enhanced T1-weighted imaging; DWI, diffusion-weighted imaging; EUS, endoscopic ultrasonography; FDG, 18F-fluorodeoxyglucose; fs, fat suppression; MRCP, magnetic resonance cholangiopancreatography; PET-CT, positron-emission tomography-computed tomography; T1WI, T1-weighted imaging; T2WI, T2-weighted imaging

**Fig. 4:**
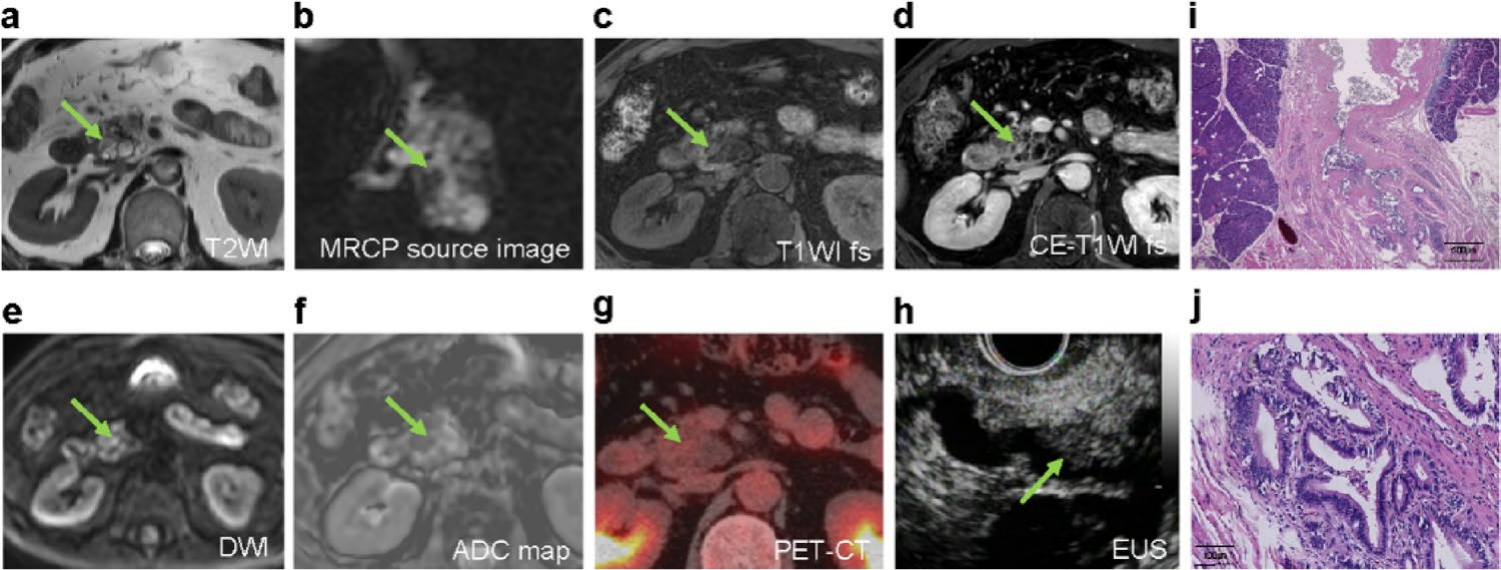
Benign enhancing lesion in a 58-year-old male. **a** T2-weighted imaging showing a cystic lesion containing a round-shaped mural nodule (green arrow). **b** Magnetic resonance cholangiopancreatography source image of the mural nodule. **c** Fat-suppressed T1-weighted imaging showing lower signal intensity than normal pancreatic parenchyma. **d** Contrast-enhanced fat-suppressed T1-weighted imaging showing enhancement of the mural nodule. **e** Diffusion-weighted imaging showing higher signal intensity than normal pancreatic parenchyma. **f** Apparent diffusion coefficient map showing lower value than normal pancreatic parenchyma. **g** Positron-emission tomography-computed tomography showing no significant ^18^F-fluorodeoxyglucose uptake (standardized uptake value maximum: 0.4). **h** Endoscopic ultrasonography confirming the presence of an enhancing solid nodule. **i** Histopathology showing intraductal papillary mucinous neoplasm with low-grade dysplasia, gastric-type epithelium forming papillary structures within existing ductal architecture (hematoxylin and eosin, scale bar: 500 μm). **j** Higher magnification demonstrating columnar epithelial cells with mildly enlarged ovoid nuclei and pyloric gland differentiation (hematoxylin and eosin, scale bar: 100 μm). Abbreviations: ADC, apparent diffusion coefficient; CE-T1WI, contrast-enhanced T1-weighted imaging; DWI, diffusion-weighted imaging; EUS, endoscopic ultrasonography; FDG, 18F-fluorodeoxyglucose; fs, fat suppression; MRCP, magnetic resonance cholangiopancreatography; PET-CT, positron-emission tomography-computed tomography; T1WI, T1-weighted imaging; T2WI, T2-weighted imaging

**Fig. 5:**
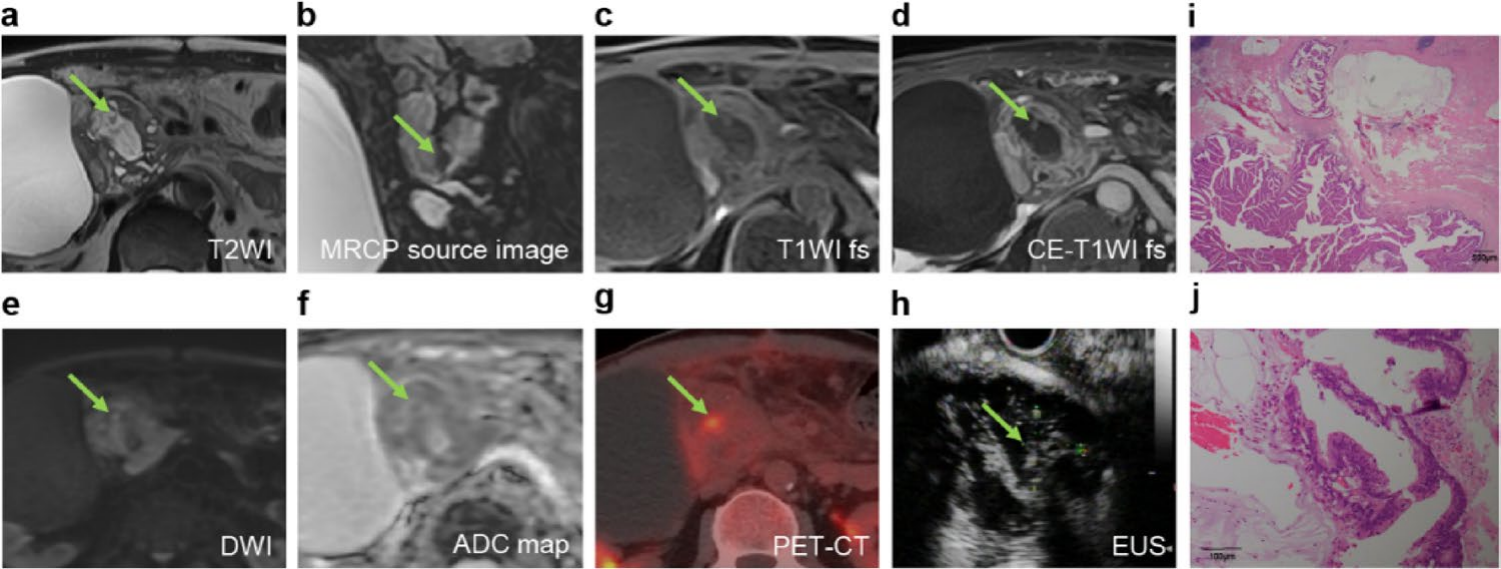
High-risk lesion in 69-year-old male. **a** T2-weighted imaging showing a cystic lesion containing a round-shaped mural nodule (green arrow). **b** Magnetic resonance cholangiopancreatography source image of the mural nodule. **c** Fat-suppressed T1-weighted imaging showing intermediate signal intensity. **d** Contrast-enhanced fat-suppressed T1-weighted imaging showing enhancement of the mural nodule. **e** Diffusion-weighted imaging showing intermediate signal intensity. **f** Apparent diffusion coefficient (ADC) map showing no significant diffusion restriction (ADC value: 1.4 × 10⁻^3^ mm^2^/s). **g** Positron-emission tomography-computed tomography showing increased ^18^F-fluorodeoxyglucose uptake (standardized uptake value maximum: 5.0). **h** Endoscopic ultrasound confirming the presence of an enhancing solid nodule. **i**) Histopathology showing intraductal papillary mucinous neoplasm with high-grade dysplasia, intestinal-type epithelium forming complex papillary structures (hematoxylin and eosin, scale bar: 500 μm). **j** Higher magnification demonstrating atypical columnar epithelium with enlarged hyperchromatic nuclei (hematoxylin and eosin, scale bar: 100 μm). Abbreviations: ADC, apparent diffusion coefficient; CE-T1WI, contrast-enhanced T1-weighted imaging; DWI, diffusion-weighted imaging; EUS, endoscopic ultrasonography; FDG, 18F-fluorodeoxyglucose; fs, fat suppression; MRCP, magnetic resonance cholangiopancreatography; PET-CT, positron-emission tomography-computed tomography; T1WI, T1-weighted imaging; T2WI, T2-weighted imaging

Table 4 presents the point estimates and 95%CIs of the odds ratios (ORs) in the multivariable models. For differentiating NELs (Pattern 1), the OR of the target sign was 6.133 (95% CI 0.505–859.11) for Reader 1 and 5.364 (95% CI 0.414–1389.35) for Reader 2. Due to the complete separation of the target sign for NELs, the OR CIs were wide. Additionally, shape, wall relationship, and cyst morphology also had high estimated ORs. Although some clinical factors exhibited wide CIs, similar to the target sign, the estimated ORs for most factors were similar between the readers. Representative cases illustrating the target sign in NEL (Fig. 3), the typical appearance of BEL (Fig. 4), and HRL (Fig. 5) demonstrate the key imaging features that aid in differentiation.

**Table 4:** Multivariable Logistic Regression Analysis for Differentiating Lesion Types

Variables	Odds ratio (95% confidence interval)			
	Pattern 1		Pattern 2	
	Reader 1	Reader 2	Reader 1	Reader 2
Intercept	0.300 (0.003–21.960)	0.082 (0.001–21.913)	14.033 (0.316–1341.773)	11.527 (0.295–1229.371)
Nodule diameter (mm)	0.797 (0.608–1.005)	0.923 (0.603–1.017)	0.887 (0.745–0.986)	0.918 (0.772–0.998)
Shape (oval)	9.431 (1.482–90.475)	21.135 (1.798–234.963)	2.194 (0.287–16.968)	4.098 (0.629–26.904)
Wall relationship (attachment)	3.075 (0.657–15.892)	2.880 (0.694–18.975)	1.234 (0.264–6.073)	1.174 (0.273–5.197)
Cyst diameter (mm)	1.043 (0.990–1.104)	1.039 (0.991–1.110)	1.005 (0.962–1.057)	1.001 (0.958–1.051)
Cyst morphology (unilocular)	28.453 (4.047–342.206)	41.237 (4.185–527.585)	4.085 (0.676–31.789)	4.555 (0.714–31.972)
MPD diameter (mm)	1.053 (0.771–1.452)	1.127 (0.777–1.545)	0.969 (0.763–1.240)	1.006 (0.798–1.262)
Age (years)	0.969 (0.910–1.033)	0.958 (0.897–1.032)	0.983 (0.930–1.031)	0.976 (0.923–1.025)
Target sign	6.133 (0.505–859.112)	5.364 (0.414–1389.353)	3.254 (0.232–463.013)	2.617 (0.177–377.719)

Pattern 1: Non-enhancing vs. enhancing lesions (primary analysis); Pattern 2: Non or benign enhancing vs. high-risk lesions (secondary analysis); Variables are shown with their indicator category in parentheses where applicable. Firth’s penalized likelihood estimation was used to address complete separation. CI, confidence interval; MPD, main pancreatic duct

## Discussion

Our study identified key MRI characteristics that differentiate NELs from mural nodules in pancreatic cystic lesions. The “target sign” was exclusively observed in NEL (41.9%) and absent in all enhancing lesions, providing 100% specificity and PPV for identifying NELs. Additionally, the shape characteristics (93.5% of NELs were oval vs. predominantly irregular shapes in true nodules) and the cyst-wall relationship (85.5% of NELs had posterior attachment) offered valuable discriminatory information. Our multiparameter model incorporating these features achieved excellent diagnostic performance, with AUCs of 0.97 for differentiating non-enhancing from enhancing lesions and 0.92 for distinguishing NELs/BELs from HRLs, significantly outperforming individual imaging features.

Our findings on morphological features align with those by Harima et al. [8] and Yamashita et al. [9], who reported that mucin plugs are mobile and settle in gravity-dependent positions. The posterior attachment pattern we observed in 85.5% of NELs supports this gravitational-effect theory. The oval-shape predominance in NELs versus irregular or villous morphology in true mural nodules corroborates observations by Fusaroli et al. [10], noting true neoplastic nodules have more complex morphological features. Our observed MRI target sign shares similarities with the EUS target sign reported by researchers [9–11, 21]. The EUS target sign is characterized by a hypoechoic center surrounded by a hyperechoic rim with smooth edges on B-mode imaging, reflecting the layered structure of mucin plugs; importantly, this sign can be identified on conventional EUS without contrast [21]. The EUS target sign is an established, valuable diagnostic feature for mucin plugs. We found a 71.3% concordance rate between MRI and EUS target signs, suggesting they probably reflect the same pathological substrate. Although CE-EUS is the gold standard for vascularity-based differentiation [8, 11, 23–25], our study demonstrated that conventional MRI provided highly accurate non-invasive differentiation.

Notably, the high PPV of the target sign is clinically important, allowing accurate identification of benign lesions and potentially eliminating the need for additional invasive examinations. The high specificity indicates a low false-positive rate, and the improved diagnostic performance of the multivariable models complements the limitations of target-sign evaluation alone, underscoring the importance of comprehensive imaging assessment. The CIs of the AUCs also narrow for both readers and across both outcome patterns, providing statistical support for the reliability of diagnostic performance. The marked difference observed between the mean diameter of NELs (6.60 ± 2.87 mm) and of HRLs (19.06 ± 14.92 mm) further supports the biological plausibility of these findings [26, 27].

**Fig. 6:**
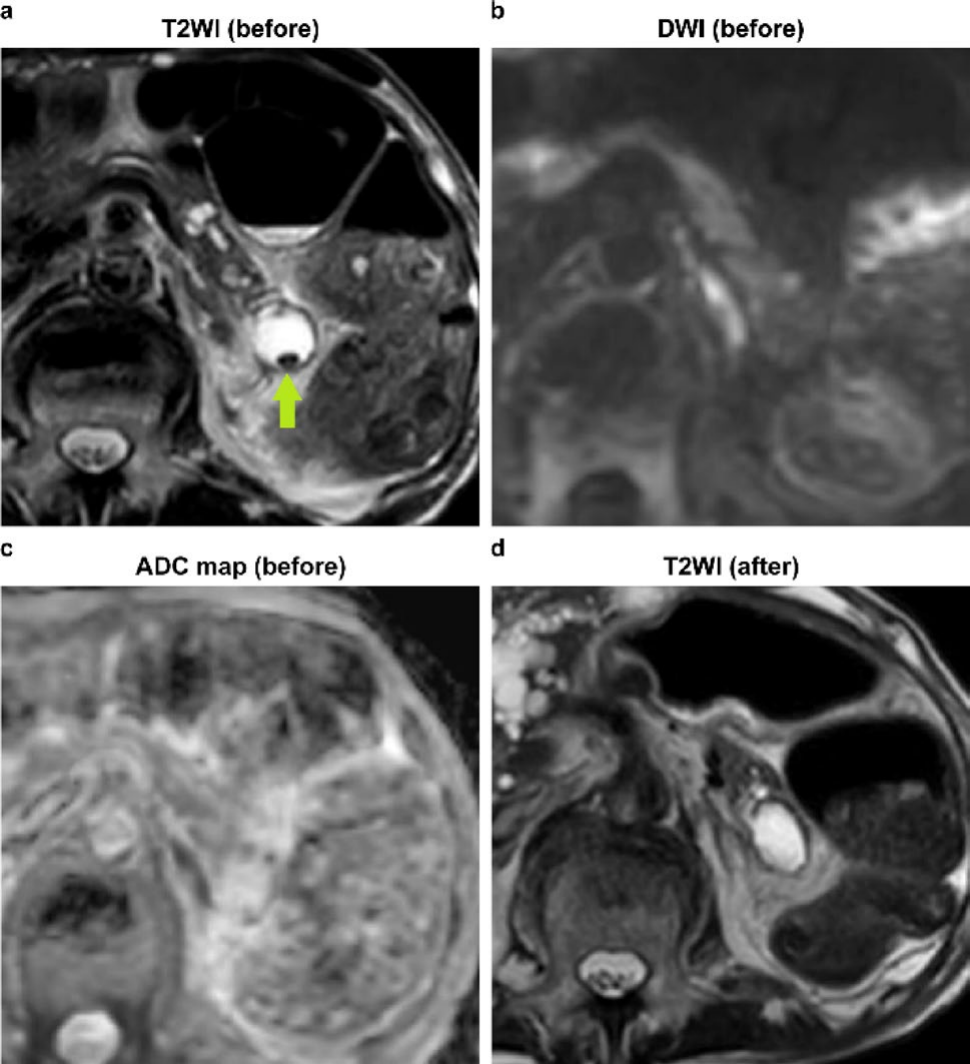
Illustrative case demonstrating the value of multiparameter assessment: Non-enhancing lesion without the target sign in a 73-year-old male patient **a** T2-weighted imaging revealed a unilocular cystic lesion in the pancreatic tail with a nodular component (arrow) exhibiting homogeneous low signal intensity without the characteristic target sign. The nodule shows posterior wall attachment with an oval shape. **b** Diffusion-weighted imaging showing low signal intensity. **c** Apparent diffusion coefficient map showing higher values than normal pancreatic parenchyma. **d** Follow-up MRI demonstrated complete disappearance of the nodule, confirming the mucin plug. Despite the absence of the target sign and an elevated serum CA19-9 level (135.1 U/mL), the morphological features yielded a low multiparameter risk score. Diffusion-weighted imaging and apparent diffusion coefficient findings further supported the diagnosis, although these parameters were not included in the predictive model. Abbreviations: ADC, apparent diffusion coefficient; CA19-9, carbohydrate antigen 19–9; DWI, diffusion-weighted imaging; T2WI, T2-weighted imaging

In the multivariable model, factors other than the target sign included nodule diameter, shape, wall relationship, cyst diameter and morphology, MPD diameter, and age. These variables were selected based on their established significance in international guidelines for pancreatic cyst management [4, 5], as well as their availability on non-contrast MRI. Overall, the model achieved excellent diagnostic performance (AUC, 0.97). Importantly, all of these parameters can be obtained from non-contrast MRI, suggesting that integration of these readily measurable features could reduce the need for contrast-enhanced imaging or invasive procedures. An illustrative case demonstrating the value of this multiparameter assessment is presented in Fig. 6, where a lesion lacking the target sign with elevated CA19-9 was correctly classified as a mucin plug based on the combined morphological features.

The DWI characteristics, low signal intensity on DWI (93.5%), and high ADC values (95.2%) in NELs versus high DWI signal (82.4%) and low ADC values (82.4%) in HRLs reflect fundamental differences in molecular diffusion. True mural nodules—particularly HRLs—exhibit restricted diffusion due to hypercellularity, whereas mucin plugs allow relatively free water-molecule movement, supporting earlier research [28–31].

Accurate differentiation between NELs and true mural nodules directly affects clinical management decisions. Pancreatic cysts with mural nodules are often considered for surgical resection as HRLs according to international guidelines [5, 32], whereas mucin plugs only require surveillance. Conventional MRI provides valuable non-invasive differentiation with high accuracy, potentially reducing unnecessary invasive procedures while ensuring appropriate management of HRLs. For radiologists, target-sign recognition and assessments of shape and position characteristics are of practical value in daily clinical interpretation. For clinicians, these findings enable confident risk stratification without the need for invasive procedures in cases of indeterminate nodular components. The “spearfishing sign” described by Umar and Chandrasekhara [21] serves as an invasive approach for distinction. Overall, our results suggest that MRI features may non-invasively provide similar diagnostic information in non-equivocal cases.

This retrospective study had limitations. First, cases were extracted from radiology reports containing specific keywords, a potential selection bias. Additionally, although strict criteria, defined as stable size ≥ 2 years or disappearance, were applied to classify NELs, surgical confirmation was not always obtained. Pathological confirmation would be ideal.

Furthermore, although Firth logistic regression was used in the multivariable model to address complete separation between the target sign and diagnostic outcome, the OR CIs for the target sign remained wide. Although the overall diagnostic performance of the model is supported by the point estimates and CIs of the AUC, uncertainty remained for the independent effects of individual factors, including the target sign. Furthermore, although excellent inter-observer agreement (κ = 0.95) was achieved among experienced radiologists, reproducibility among less-experienced radiologists remains unclear. Finally, these single-center study findings may reflect center-specific practices, potentially limiting generalizability.

This study demonstrated that non-contrast MRI features—particularly the target sign, shape characteristics, and their cyst-wall relationship—can reliably differentiate NELs from true mural nodules in pancreatic cystic lesions. Notably, the target sign showed 100% specificity for mucin plugs, and the multiparameter model achieved excellent diagnostic performance (AUC of 0.97) without contrast-enhanced imaging or invasive procedures. These findings support the clinical utility of abbreviated non-contrast MRI protocols for surveillance of pancreatic cystic lesions, potentially reducing patient burden, costs, and risks associated with contrast administration. Future prospective multicenter studies with larger cohorts are needed to validate these findings across diverse clinical settings and patient populations.

## Supplementary Information

Below is the link to the electronic supplementary material.

Supplementary Material 1
