# Supplementary material for: Non-contrast MRI features of mucin plugs and mural nodules in pancreatic cystic lesions
Source: Jpn J Radiol. 2026 Mar 31;44(7):1189–99. doi: 10.1007/s11604-026-01975-x (PMC13315461; doi:10.1007/s11604-026-01975-x)
Supplement: Supplementary file 1 — Supplementary Material 1 [file 11604_2026_1975_MOESM1_ESM.docx]

**Non-contrast MRI Features of Mucin Plugs and Mural Nodules in Pancreatic Cystic Lesions**

Journal name: Japanese Journal of Radiology

Hideyuki Fukui, MD¹*, Atsushi Nakamoto, MD, PhD¹, Hiromitsu Onishi, MD, PhD¹, Takashi Ota, MD, PhD¹, Yasunari Fukuda, MD, PhD², ³ Toru Honda, MD, PhD¹, Sakiko Ueno, MD¹, Feier Ding, MD¹, Masahiro Umezu, MD², Daisaku Yamada, MD, PhD², Hidetoshi Eguchi, MD, PhD², Noriyuki Tomiyama, MD, PhD¹

^1^ Department of Diagnostic and Interventional Radiology, The University of Osaka Graduate School of Medicine

^2^ Department of Gastroenterological Surgery, The University of Osaka Graduate School of Medicine

^3^ Department of Surgery, Osaka International Cancer Institute

*Corresponding author

Hideyuki Fukui

Department of Diagnostic and Interventional Radiology, The University of Osaka Graduate School of Medicine,

D1, 2-2, Yamadaoka, Suita, Osaka 565-0871 JAPAN

Tel: +81-6-6879-3434, Fax: +81-6-6879-3439

Email: hidefukui@infoseek.jp

ORCID: 0000-0002-6330-2482

**Supplemental Table E1. MRI Systems and Parameters**

| **Parameter** | **Specifications** |
| --- | --- |
| **Systems** | 3.0-T: SIGNA Architect, Discovery 750, Discovery 750w, Signa HDxt (GE Healthcare); Achieva, Achieva dStream (Philips); Centurian (Canon) 1.5T: Ingenia (Philips) |
| **Coils** | Phased-array body coils |
| **T2-weighted imaging** | Fast spin-echo sequences (SSFSE, ssh, FACE) - Slice thickness: 5 mm - Interslice gap: 0.5–2.0 mm - Matrix size: 224 × 224–320 × 256 - Pixel bandwidth: 244–651 Hz/pixel - Field of view: 385 × 385 mm (axial) |
| **T1-weighted imaging** | Dual gradient-echo sequences (LAVA Flex, mDixon, WFS) - Slice thickness: 4–5 mm - Interslice gap: −1 to 0 mm - Matrix size: 160 × 192–320 × 192 - Pixel bandwidth: 558–1302 Hz/pixel - Field of view: 385 × 270 mm (axial) |
| **MRCP source images** | Breath-hold/respiratory-triggered T2-weighted sequences - Slice thickness: 1.6–3.0 mm - Interslice gap: −1.5 to −0.8 mm - Matrix size: 256 × 185 to 512 × 320 - Pixel bandwidth: 325–1335 Hz/pixel - Field of view: 385–408 × 320 mm (coronal) |
| **Balanced steady-state** | FIESTA, bTFE, SSFP sequences - Slice thickness: 5 mm - Interslice gap: −1.0 to −0.5 mm - Matrix size: 192 × 288–208 × 224 - Pixel bandwidth: 244–2100 Hz/pixel - Field of view: 385–408 × 385–408 mm (coronal) |
| **Diffusion-weighted** | Single-shot echo-planar sequences - b values: 600, 800, 1000 s/mm² - Slice thickness: 5 mm - Interslice gap: 2 mm - Matrix size: 96 × 84–128 × 128 - Pixel bandwidth: 1953–4319 Hz/pixel - Field of view: 385 × 290 mm (axial) |
| **Post-contrast T1-weighted imaging** | 3D gradient-echo sequences - Slice thickness: 4 mm - Interslice gap: −2 mm - Matrix size: 320 × 192 - Pixel bandwidth: 325 Hz/pixel - Field of view: 385 × 385 mm (axial) |

**Supplemental Table E2: Cyst Characteristics by Diagnostic Group (Reader 2)**

| **Parameter** | **Category** | **Nonenhancing Lesions** | **Benign Enhancing Lesions** | **High-Risk Lesions** | **Total** |
| --- | --- | --- | --- | --- | --- |
| **MRCP Balance T2WI** | High | 57 (100.0%) | 22 (100.0%) | 17 (100.0%) | 96 (100.0%) |
|  | Intermediate | 0 (0.0%) | 0 (0.0%) | 0 (0.0%) | 0 (0.0%) |
|  | Low | 0 (0.0%) | 0 (0.0%) | 0 (0.0%) | 0 (0.0%) |
|  | **Subtotal** | **57 (100.0%)** | **22 (100.0%)** | **17 (100.0%)** | **96 (100.0%)** |
| **T1WIfs** | High | 1 (1.8%) | 1 (4.5%) | 0 (0.0%) | 2 (2.1%) |
|  | Intermediate | 0 (0.0%) | 0 (0.0%) | 0 (0.0%) | 0 (0.0%) |
|  | Low | 56 (98.2%) | 21 (95.5%) | 17 (100.0%) | 94 (97.9%) |
|  | **Subtotal** | **57 (100.0%)** | **22 (100.0%)** | **17 (100.0%)** | **96 (100.0%)** |
| **DWI** | High | 1 (1.8%) | 0 (0.0%) | 0 (0.0%) | 1 (1.0%) |
|  | Intermediate | 0 (0.0%) | 1 (4.5%) | 0 (0.0%) | 1 (1.0%) |
|  | Low | 56 (98.2%) | 21 (95.5%) | 17 (100.0%) | 94 (97.9%) |
|  | **Subtotal** | **57 (100.0%)** | **22 (100.0%)** | **17 (100.0%)** | **96 (100.0%)** |
| **ADC** | High | 57 (100.0%) | 22 (100.0%) | 17 (100.0%) | 96 (100.0%) |
|  | Intermediate | 0 (0.0%) | 0 (0.0%) | 0 (0.0%) | 0 (0.0%) |
|  | Low | 0 (0.0%) | 0 (0.0%) | 0 (0.0%) | 0 (0.0%) |
|  | **Subtotal** | **57 (100.0%)** | **22 (100.0%)** | **17 (100.0%)** | **96 (100.0%)** |

Values are presented as n (%). Abbreviations: **MPD**, main pancreatic duct; **MRCP**, magnetic resonance cholangiopancreatography; **T2WI**, T2-weighted imaging; **T1WIfs**, fat-suppressed T1-weighted imaging; **DWI**, diffusion-weighted imaging; **ADC**, apparent diffusion coefficient.

**Supplemental Table E3: Nodule Characteristics and MRI Features by Diagnostic Group (Reader 2)**

| **MRI Sequence** | **Category** | **Nonenhancing Lesions** | **Benign Enhancing Lesions** | **High-Risk Lesions** | **Total** |
| --- | --- | --- | --- | --- | --- |
| **MRCP/Balance T2WI** | High | 0 (0.0%) | 2 (9.1%) | 0 (0.0%) | 2 (2.0%) |
|  | Intermediate | 6 (9.7%) | 8 (36.4%) | 14 (82.4%) | 28 (27.7%) |
|  | Low | 30 (48.4%) | 12 (54.5%) | 3 (17.6%) | 45 (44.6%) |
|  | Target | 26 (41.9%) | 0 (0.0%) | 0 (0.0%) | 26 (25.7%) |
|  | **Subtotal** | **62 (100.0%)** | **22 (100.0%)** | **17 (100.0%)** | **101 (100.0%)** |
| **T1WIfs** | High | 1 (1.6%) | 0 (0.0%) | 0 (0.0%) | 1 (1.0%) |
|  | Intermediate | 1 (1.6%) | 3 (13.6%) | 4 (23.5%) | 8 (7.9%) |
|  | Low | 58 (93.5%) | 19 (86.4%) | 13 (76.5%) | 90 (89.1%) |
|  | Target | 2 (3.2%) | 0 (0.0%) | 0 (0.0%) | 2 (2.0%) |
|  | **Subtotal** | **62 (100.0%)** | **22 (100.0%)** | **17 (100.0%)** | **101 (100.0%)** |
| **DWI** | High | 2 (3.2%) | 11 (50.0%) | 14 (82.4%) | 27 (26.7%) |
|  | Intermediate | 2 (3.2%) | 4 (18.2%) | 1 (5.9%) | 7 (6.9%) |
|  | Low | 58 (93.5%) | 7 (31.8%) | 2 (11.8%) | 67 (66.3%) |
|  | **Subtotal** | **62 (100.0%)** | **22 (100.0%)** | **17 (100.0%)** | **101 (100.0%)** |
| **ADC** | High | 59 (95.2%) | 9 (40.9%) | 3 (17.6%) | 71 (70.3%) |
|  | Intermediate | 0 (0.0%) | 2 (9.1%) | 1 (5.9%) | 3 (3.0%) |
|  | Low | 3 (4.8%) | 11 (50.0%) | 13 (76.5%) | 27 (26.7%) |
|  | **Subtotal** | **62 (100.0%)** | **22 (100.0%)** | **17 (100.0%)** | **101 (100.0%)** |

Values are presented as n (%). Abbreviations: **MPD**, main pancreatic duct; **MRCP**, magnetic resonance cholangiopancreatography; **T2WI**, T2-weighted imaging; **T1WIfs**, fat-suppressed T1-weighted imaging; **DWI**, diffusion-weighted imaging; **ADC**, apparent diffusion coefficient.
